# *rac*-2,2′-Bis(diphenyl­phosphan­yl)-1,1′-binaphth­yl: a racemic diphosphine ligand

**DOI:** 10.1107/S1600536812025603

**Published:** 2012-06-13

**Authors:** Feng Niu, Wenxiang Chai, Li Song, Mengbo Zhou, Jiaping Liang

**Affiliations:** a College of Materials Science and Engineering, China Jiliang University, Hangzhou 310018, People’s Republic of China; b Department of Chemistry, Key Laboratory of Advanced Textile Materials and Manufacturing Technology of the Education Ministry, Zhejiang Sci-Tech University, Hangzhou 310018, People’s Republic of China

## Abstract

The asymmetric unit of the title compound, C_44_H_32_P_2_, conventionally abbreviated BINAP, is one half of the complete chiral BINAP mol­ecule, which adopts a *C*2 crystallographic point-group symmetry with a twofold axis splitting the mol­ecule in two identical halves; a center of symmetry between mol­ecules further determines the racemic pairs. There are no obvious supra­molecular inter­actions between adjacent BINAP mol­ecules.

## Related literature

For applications of triaryl­phosphine ligands in various catalytic reactions, see: Doherty *et al.* (2012 ▶); Uemura *et al.* (2012 ▶); Onodera *et al.* (2012 ▶); Lin *et al.* (2012 ▶). For applications of 2,2′-bis­(diphenyl­phosphan­yl)-1,1′-binaphthyl (BINAP) as a chiral catalyst in various asymmetric catalysed reactions, see: Kojima & Mikami (2012 ▶); Aikawa *et al.* (2011 ▶); Ge & Hartwig (2011 ▶); Moran *et al.* (2011 ▶). For similar diphosphine ligands, see: Kassube *et al.* (2008 ▶); Fawcett *et al.* (2005 ▶); Wu *et al.* (2004 ▶). For the related crystal structure of the (S)-enanti­omer (*S*)-(−)-2,2′-bis­(diphenyl­phosphan­yl)-1,1′-binaphthyl, see: Jones *et al.* (2003 ▶).
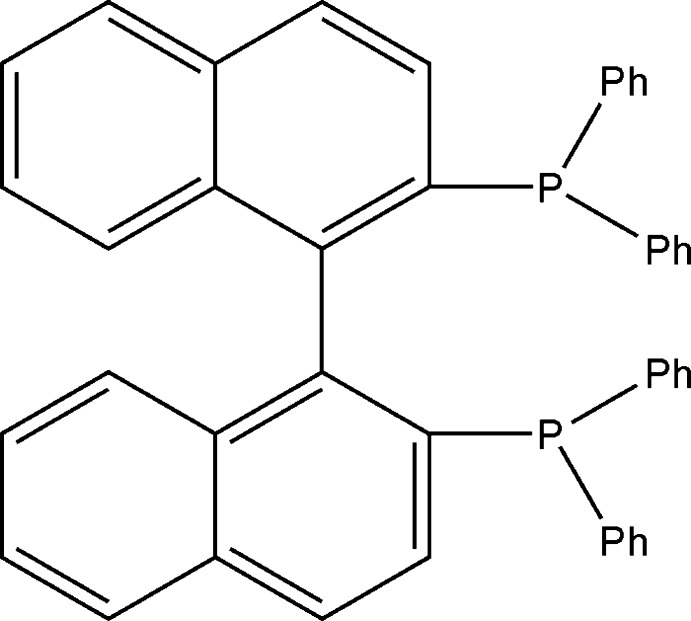


## Experimental

### 

#### Crystal data

C_44_H_32_P_2_
*M* *_r_* = 622.64
Monoclinic, 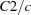

*a* = 19.6120 (8) Å
*b* = 9.2008 (3) Å
*c* = 19.1240 (9) Å
β = 107.904 (5)°
*V* = 3283.7 (2) Å^3^
*Z* = 4
Mo *K*α radiation
μ = 0.16 mm^−1^
*T* = 293 K
0.29 × 0.23 × 0.20 mm

#### Data collection

Oxford Diffraction Xcalibur Gemini ultra diffractometer
Absorption correction: multi-scan (*ABSCOR*; Higashi, 1995 ▶) *T*_min_ = 0.954, *T*_max_ = 0.968
6234 measured reflections
3052 independent reflections
2314 reflections with *I* > 2σ(*I*)
*R*_int_ = 0.026

#### Refinement

*R*[*F* ^2^ > 2σ(*F* ^2^)] = 0.042
*wR*(*F* ^2^) = 0.099
*S* = 1.03
3052 reflections
208 parameters
H-atom parameters constrained
Δρ_max_ = 0.22 e Å^−3^
Δρ_min_ = −0.22 e Å^−3^

### 

Data collection: *CrysAlis PRO* (Oxford Diffraction, 2011 ▶); cell refinement: *CrysAlis PRO*; data reduction: *CrysAlis PRO*; program(s) used to solve structure: *SHELXS97* (Sheldrick, 2008 ▶); program(s) used to refine structure: *SHELXL97* (Sheldrick, 2008 ▶); molecular graphics: *SHELXTL* (Sheldrick, 2008 ▶); software used to prepare material for publication: *SHELXTL*.

## Supplementary Material

Crystal structure: contains datablock(s) I, global. DOI: 10.1107/S1600536812025603/bg2467sup1.cif

Structure factors: contains datablock(s) I. DOI: 10.1107/S1600536812025603/bg2467Isup2.hkl

Supplementary material file. DOI: 10.1107/S1600536812025603/bg2467Isup3.cml

Additional supplementary materials:  crystallographic information; 3D view; checkCIF report

## Figures and Tables

**Fig. 1.**
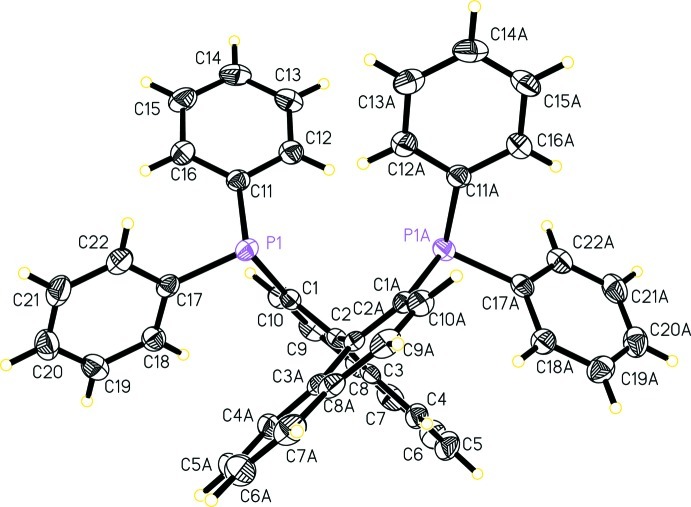
A view of the structure of I, showing the atom-numbering scheme. Displacement ellipsoids are drawn at the 30% probabilithy level, and H atoms shown as small spheres of arbitrary radii.

**Fig. 2.**
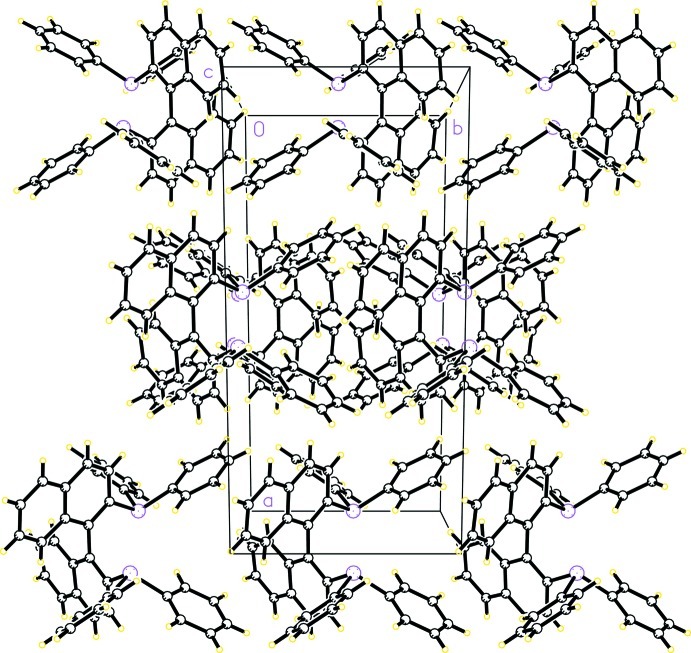
The packing diagram of I, viewed along the c-direction.

## supplementary crystallographic information

### Comment

In the past decades, the triarylphosphine ligands have been well known for their high catalytic activity and high selectivity, and have thus been widely used in various types of reactions, *e.g.*, asymmetrical hydrogen catalysis, Buchwald-Hartwig C—N and C—O formations and Suzuki coupling reactions, *etc* (Doherty *et al.* 2012; Uemura *et al.* 2012; Onodera *et al.* 2012; Lin *et al.* 2012). Among them, 2,2'-Bis(diphenylphosphanyl)-1,1'-binaphthyl (BINAP), a diphosphine ligand, is well known for its chirality, and has been used as chiral catalyst in various asymmetry catalyzed reaction (Kojima *et al.* 2012; Aikawa *et al.* 2011; Ge *et al.* 2011; Moran *et al.* 2011). Previously, Jones *M*. D. *etc* reported the crystal structure of the (S)-enantiomer (*S*)-(-)-2,2'-Bis(diphenylphosphanyl)-1,1'-binaphthyl (Jones *et al.* 2003). But the crystal structure of the racemic BINAP crystal had not been reported so far. Here we report the crystal structure of the racemic 2,2'-Bis(diphenylphosphanyl)-1,1'-binaphthyl, which may provide some useful structural information for its chiral separation and high catalytic activity / selectivity.

The title compound crystallizes in the centrosymmetric *C*2/*c* space group. The asymmetric unit is one half of complete chiral BINAP molecule, the symmetry related part being generated by a twofold rotational axis running along *b* and across the middle of two moities in the molecule (Fig 1). This structure is similar to the previously reported one for the (S)-enantiomer (*S*)-(-)-2,2'-Bis(diphenylphosphanyl)-1,1'-binaphthyl (Jones *et al.* 2003). In the present case, however, there are inversion centers relating molecules into racemic pairs. No obvious supramolecular interactions are present in the crystal structure of (1), which packing diagram is shown in Fig 2.

### Experimental

Crystals of the title compound were obtained by recrystalization of racemic 2,2'-Bis(diphenylphosphanyl)-1,1'-binaphthyl, obtained from Acros Organics as a commerical material: the racemic powders were dissolved in dichloromethane, and after being filtrated some isopropyl alcohol was layered on the resulting solution. After several days, a crop of colorless crystals of (I) were abtained, fron where specimens suitable for single-crystal X-ray diffraction were selected.

### Refinement

All aromatoc hydrogen atoms were added at calculated positions (C-H: 0.93Å) and refined using a riding model with U(H)iso = 1.2× U(C)equiv.

### Crystal data

**Table:**

C_44_H_32_P_2_	*F*(000) = 1304
*M**_r_* = 622.64	*D*_x_ = 1.259 Mg m^−^^3^
Monoclinic, *C*2/*c*	Mo *K*α radiation, λ = 0.71070 Å
Hall symbol: -C 2yc	Cell parameters from 2381 reflections
*a* = 19.6120 (8) Å	θ = 3.5–29.5°
*b* = 9.2008 (3) Å	µ = 0.16 mm^−^^1^
*c* = 19.1240 (9) Å	*T* = 293 K
β = 107.904 (5)°	Column, colourless
*V* = 3283.7 (2) Å^3^	0.29 × 0.23 × 0.20 mm
*Z* = 4	

### Data collection

**Table:**

Oxford Diffraction Xcalibur Gemini ultra diffractometer	3052 independent reflections
Radiation source: Enhance (Mo) X-ray Source	2314 reflections with *I* > 2σ(*I*)
Graphite monochromator	*R*_int_ = 0.026
Detector resolution: 10.3592 pixels mm^-1^	θ_max_ = 25.5°, θ_min_ = 3.6°
ω scans	*h* = −19→23
Absorption correction: multi-scan (*ABSCOR*; Higashi, 1995)	*k* = −9→11
*T*_min_ = 0.954, *T*_max_ = 0.968	*l* = −20→23
6234 measured reflections	

### Refinement

**Table:**

Refinement on *F*^2^	Primary atom site location: structure-invariant direct methods
Least-squares matrix: full	Secondary atom site location: difference Fourier map
*R*[*F*^2^ > 2σ(*F*^2^)] = 0.042	Hydrogen site location: inferred from neighbouring sites
*wR*(*F*^2^) = 0.099	H-atom parameters constrained
*S* = 1.03	*w* = 1/[σ^2^(*F*_o_^2^) + (0.0387*P*)^2^ + 1.4219*P*] where *P* = (*F*_o_^2^ + 2*F*_c_^2^)/3
3052 reflections	(Δ/σ)_max_ = 0.001
208 parameters	Δρ_max_ = 0.22 e Å^−^^3^
0 restraints	Δρ_min_ = −0.22 e Å^−^^3^

### Special details

**Table:**

Geometry. All e.s.d.'s (except the e.s.d. in the dihedral angle between two l.s. planes) are estimated using the full covariance matrix. The cell e.s.d.'s are taken into account individually in the estimation of e.s.d.'s in distances, angles and torsion angles; correlations between e.s.d.'s in cell parameters are only used when they are defined by crystal symmetry. An approximate (isotropic) treatment of cell e.s.d.'s is used for estimating e.s.d.'s involving l.s. planes.
Refinement. Refinement of *F*^2^ against ALL reflections. The weighted *R*-factor *wR* and goodness of fit *S* are based on *F*^2^, conventional *R*-factors *R* are based on *F*, with *F* set to zero for negative *F*^2^. The threshold expression of *F*^2^ > σ(*F*^2^) is used only for calculating *R*-factors(gt) *etc*. and is not relevant to the choice of reflections for refinement. *R*-factors based on *F*^2^ are statistically about twice as large as those based on *F*, and *R*- factors based on ALL data will be even larger.

### Fractional atomic coordinates and isotropic or equivalent isotropic displacement parameters (Å^2^)

**Table:**

	*x*	*y*	*z*	*U*_iso_*/*U*_eq_	
P1	0.43987 (2)	0.96498 (5)	0.13904 (3)	0.03969 (16)	
C1	0.41174 (9)	1.08276 (19)	0.20272 (9)	0.0348 (4)	
C2	0.46167 (8)	1.17151 (17)	0.25019 (9)	0.0316 (4)	
C3	0.44162 (9)	1.26196 (19)	0.30150 (9)	0.0368 (4)	
C4	0.49012 (11)	1.3589 (2)	0.34918 (11)	0.0492 (5)	
H4	0.5364	1.3677	0.3463	0.059*	
C5	0.47017 (14)	1.4398 (3)	0.39936 (12)	0.0680 (7)	
H5	0.5027	1.5037	0.4299	0.082*	
C6	0.40094 (15)	1.4271 (3)	0.40508 (13)	0.0718 (7)	
H6	0.3882	1.4804	0.4405	0.086*	
C7	0.35255 (13)	1.3379 (2)	0.35949 (13)	0.0609 (6)	
H7	0.3066	1.3314	0.3636	0.073*	
C8	0.37053 (10)	1.2543 (2)	0.30552 (11)	0.0434 (5)	
C9	0.32087 (10)	1.1626 (2)	0.25612 (12)	0.0489 (5)	
H9	0.2740	1.1582	0.2576	0.059*	
C10	0.34033 (9)	1.0807 (2)	0.20647 (11)	0.0450 (5)	
H10	0.3063	1.0219	0.1742	0.054*	
C11	0.38645 (9)	0.8015 (2)	0.13995 (11)	0.0422 (5)	
C12	0.39753 (10)	0.7322 (2)	0.20704 (12)	0.0524 (5)	
H12	0.4278	0.7746	0.2494	0.063*	
C13	0.36481 (11)	0.6022 (2)	0.21238 (13)	0.0581 (6)	
H13	0.3728	0.5583	0.2580	0.070*	
C14	0.32063 (12)	0.5375 (2)	0.15075 (15)	0.0634 (6)	
H14	0.2987	0.4493	0.1543	0.076*	
C15	0.30870 (12)	0.6029 (2)	0.08377 (15)	0.0686 (7)	
H15	0.2786	0.5588	0.0418	0.082*	
C16	0.34129 (11)	0.7350 (2)	0.07787 (12)	0.0585 (6)	
H16	0.3327	0.7787	0.0321	0.070*	
C17	0.39622 (9)	1.0482 (2)	0.04936 (10)	0.0404 (4)	
C18	0.35445 (10)	1.1731 (2)	0.03891 (11)	0.0472 (5)	
H18	0.3428	1.2137	0.0783	0.057*	
C19	0.32997 (11)	1.2376 (2)	−0.02968 (12)	0.0573 (6)	
H19	0.3017	1.3206	−0.0359	0.069*	
C20	0.34691 (12)	1.1805 (3)	−0.08887 (12)	0.0617 (6)	
H20	0.3309	1.2253	−0.1346	0.074*	
C21	0.38763 (12)	1.0571 (3)	−0.07949 (12)	0.0611 (6)	
H21	0.3987	1.0171	−0.1193	0.073*	
C22	0.41240 (11)	0.9914 (2)	−0.01138 (12)	0.0529 (5)	
H22	0.4403	0.9079	−0.0059	0.063*	

### Atomic displacement parameters (Å^2^)

**Table:**

	*U*^11^	*U*^22^	*U*^33^	*U*^12^	*U*^13^	*U*^23^
P1	0.0373 (3)	0.0378 (3)	0.0424 (3)	−0.0027 (2)	0.0100 (2)	−0.0060 (2)
C1	0.0349 (9)	0.0343 (9)	0.0348 (10)	0.0003 (8)	0.0099 (8)	0.0037 (8)
C2	0.0341 (9)	0.0289 (9)	0.0321 (10)	0.0028 (7)	0.0106 (7)	0.0057 (7)
C3	0.0449 (10)	0.0330 (9)	0.0338 (10)	0.0092 (8)	0.0139 (8)	0.0087 (8)
C4	0.0547 (12)	0.0490 (12)	0.0414 (12)	0.0092 (10)	0.0112 (9)	−0.0061 (10)
C5	0.0838 (17)	0.0663 (15)	0.0498 (14)	0.0151 (13)	0.0146 (12)	−0.0185 (12)
C6	0.099 (2)	0.0709 (16)	0.0539 (15)	0.0304 (15)	0.0354 (14)	−0.0065 (13)
C7	0.0728 (15)	0.0642 (14)	0.0584 (15)	0.0275 (13)	0.0386 (13)	0.0129 (12)
C8	0.0505 (11)	0.0415 (11)	0.0445 (11)	0.0136 (9)	0.0240 (9)	0.0132 (9)
C9	0.0374 (10)	0.0544 (12)	0.0615 (14)	0.0078 (10)	0.0248 (10)	0.0153 (11)
C10	0.0340 (10)	0.0461 (11)	0.0538 (13)	−0.0030 (9)	0.0120 (9)	0.0049 (10)
C11	0.0372 (10)	0.0371 (10)	0.0491 (12)	0.0027 (8)	0.0086 (9)	−0.0020 (9)
C12	0.0511 (12)	0.0467 (12)	0.0545 (14)	−0.0014 (10)	0.0091 (10)	0.0005 (10)
C13	0.0571 (13)	0.0457 (12)	0.0712 (16)	0.0027 (11)	0.0193 (12)	0.0126 (12)
C14	0.0590 (14)	0.0376 (11)	0.094 (2)	−0.0063 (11)	0.0240 (13)	0.0040 (13)
C15	0.0681 (15)	0.0488 (13)	0.0762 (18)	−0.0184 (12)	0.0035 (13)	−0.0153 (13)
C16	0.0642 (13)	0.0484 (12)	0.0534 (14)	−0.0097 (11)	0.0040 (11)	−0.0036 (11)
C17	0.0399 (10)	0.0405 (10)	0.0417 (11)	−0.0104 (9)	0.0138 (8)	−0.0061 (9)
C18	0.0494 (11)	0.0485 (12)	0.0453 (12)	−0.0019 (10)	0.0168 (9)	0.0004 (10)
C19	0.0561 (12)	0.0542 (13)	0.0612 (15)	−0.0013 (11)	0.0176 (11)	0.0115 (12)
C20	0.0645 (14)	0.0730 (16)	0.0449 (14)	−0.0165 (13)	0.0130 (11)	0.0066 (12)
C21	0.0715 (15)	0.0713 (16)	0.0437 (13)	−0.0170 (13)	0.0223 (11)	−0.0128 (12)
C22	0.0576 (13)	0.0511 (12)	0.0516 (13)	−0.0071 (10)	0.0191 (10)	−0.0106 (10)

### Geometric parameters (Å, º)

**Table:**

P1—C17	1.833 (2)	C11—C12	1.389 (3)
P1—C11	1.8364 (19)	C12—C13	1.376 (3)
P1—C1	1.8370 (18)	C12—H12	0.9300
C1—C2	1.380 (2)	C13—C14	1.366 (3)
C1—C10	1.424 (2)	C13—H13	0.9300
C2—C3	1.431 (2)	C14—C15	1.369 (3)
C2—C2^i^	1.506 (3)	C14—H14	0.9300
C3—C4	1.414 (3)	C15—C16	1.394 (3)
C3—C8	1.421 (2)	C15—H15	0.9300
C4—C5	1.363 (3)	C16—H16	0.9300
C4—H4	0.9300	C17—C18	1.389 (3)
C5—C6	1.400 (3)	C17—C22	1.397 (3)
C5—H5	0.9300	C18—C19	1.384 (3)
C6—C7	1.351 (3)	C18—H18	0.9300
C6—H6	0.9300	C19—C20	1.378 (3)
C7—C8	1.416 (3)	C19—H19	0.9300
C7—H7	0.9300	C20—C21	1.368 (3)
C8—C9	1.410 (3)	C20—H20	0.9300
C9—C10	1.356 (3)	C21—C22	1.382 (3)
C9—H9	0.9300	C21—H21	0.9300
C10—H10	0.9300	C22—H22	0.9300
C11—C16	1.386 (3)		
			
C17—P1—C11	104.33 (8)	C12—C11—P1	117.20 (14)
C17—P1—C1	102.99 (8)	C13—C12—C11	121.6 (2)
C11—P1—C1	100.87 (8)	C13—C12—H12	119.2
C2—C1—C10	119.00 (16)	C11—C12—H12	119.2
C2—C1—P1	119.23 (12)	C14—C13—C12	120.1 (2)
C10—C1—P1	121.72 (14)	C14—C13—H13	119.9
C1—C2—C3	120.43 (15)	C12—C13—H13	119.9
C1—C2—C2^i^	120.30 (15)	C13—C14—C15	119.7 (2)
C3—C2—C2^i^	119.25 (15)	C13—C14—H14	120.1
C4—C3—C8	118.24 (17)	C15—C14—H14	120.1
C4—C3—C2	122.49 (16)	C14—C15—C16	120.6 (2)
C8—C3—C2	119.27 (16)	C14—C15—H15	119.7
C5—C4—C3	121.1 (2)	C16—C15—H15	119.7
C5—C4—H4	119.4	C11—C16—C15	120.2 (2)
C3—C4—H4	119.4	C11—C16—H16	119.9
C4—C5—C6	120.3 (2)	C15—C16—H16	119.9
C4—C5—H5	119.8	C18—C17—C22	117.69 (19)
C6—C5—H5	119.8	C18—C17—P1	124.43 (15)
C7—C6—C5	120.4 (2)	C22—C17—P1	117.50 (15)
C7—C6—H6	119.8	C19—C18—C17	120.55 (19)
C5—C6—H6	119.8	C19—C18—H18	119.7
C6—C7—C8	121.2 (2)	C17—C18—H18	119.7
C6—C7—H7	119.4	C20—C19—C18	120.9 (2)
C8—C7—H7	119.4	C20—C19—H19	119.5
C9—C8—C7	122.56 (19)	C18—C19—H19	119.5
C9—C8—C3	118.73 (17)	C21—C20—C19	119.1 (2)
C7—C8—C3	118.71 (19)	C21—C20—H20	120.4
C10—C9—C8	121.12 (17)	C19—C20—H20	120.4
C10—C9—H9	119.4	C20—C21—C22	120.6 (2)
C8—C9—H9	119.4	C20—C21—H21	119.7
C9—C10—C1	121.37 (18)	C22—C21—H21	119.7
C9—C10—H10	119.3	C21—C22—C17	121.1 (2)
C1—C10—H10	119.3	C21—C22—H22	119.5
C16—C11—C12	117.79 (18)	C17—C22—H22	119.5
C16—C11—P1	124.71 (16)		

Symmetry code: (i) −*x*+1, *y*, −*z*+1/2.
